# Supplementary material for: Distinct Role of CD11b+Ly6G−Ly6C− Myeloid-Derived Cells on the Progression of the Primary Tumor and Therapy-Associated Recurrent Brain Tumor
Source: Cells. 2019 Dec 24;9(1):51. doi: 10.3390/cells9010051 (PMC7016541; doi:10.3390/cells9010051)

Figure S1

A

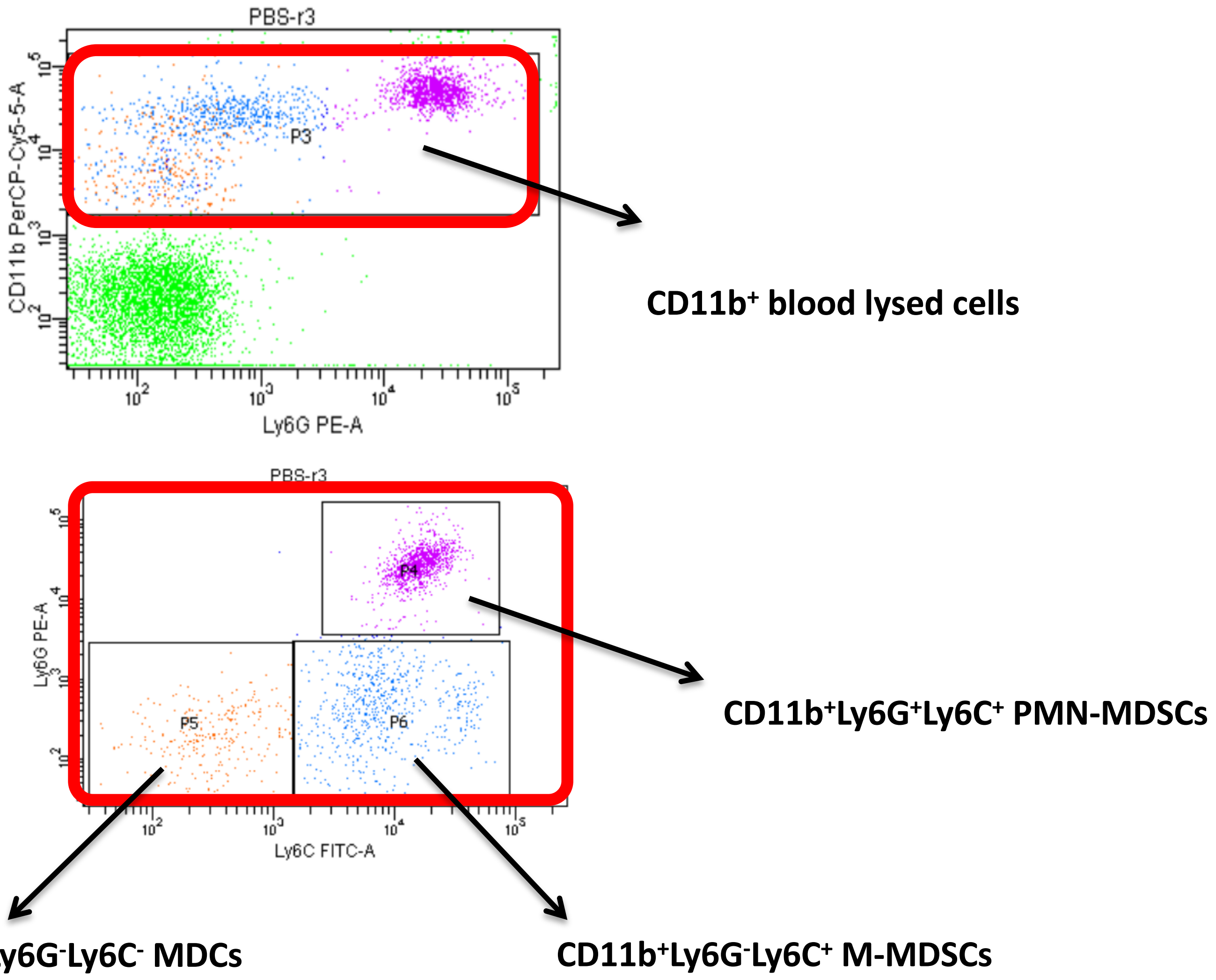
PBS-r3
CD11b PerCP-Cy5-5-A
P3
Ly6G PE-A
CD11b⁺ blood lysed cells
PBS-r3
Ly6G PE-A
P4
P5
P6
Ly6C FITC-A
CD11b⁺Ly6G⁺Ly6C⁺ PMN-MDSCs
CD11b⁺Ly6G⁻Ly6C⁻ MDCs
CD11b⁺Ly6G⁻Ly6C⁺ M-MDSCs

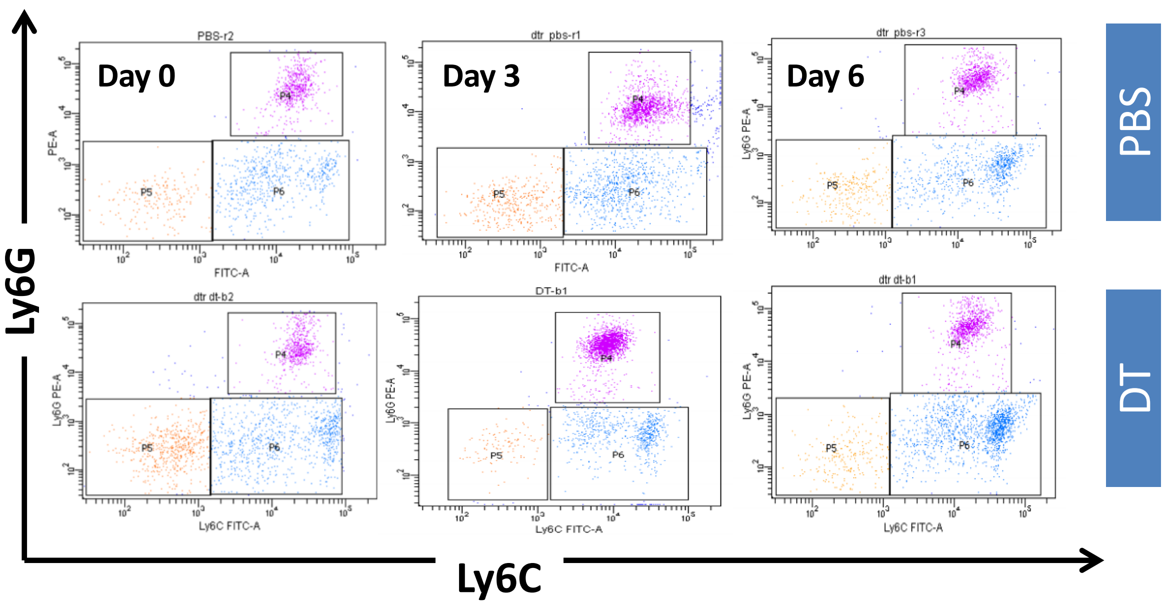
Day 0
Day 3
Day 6
PBS
DT
Ly6G
Ly6C
P4
P5
P6
Ly6C FITC-A

Figure S2

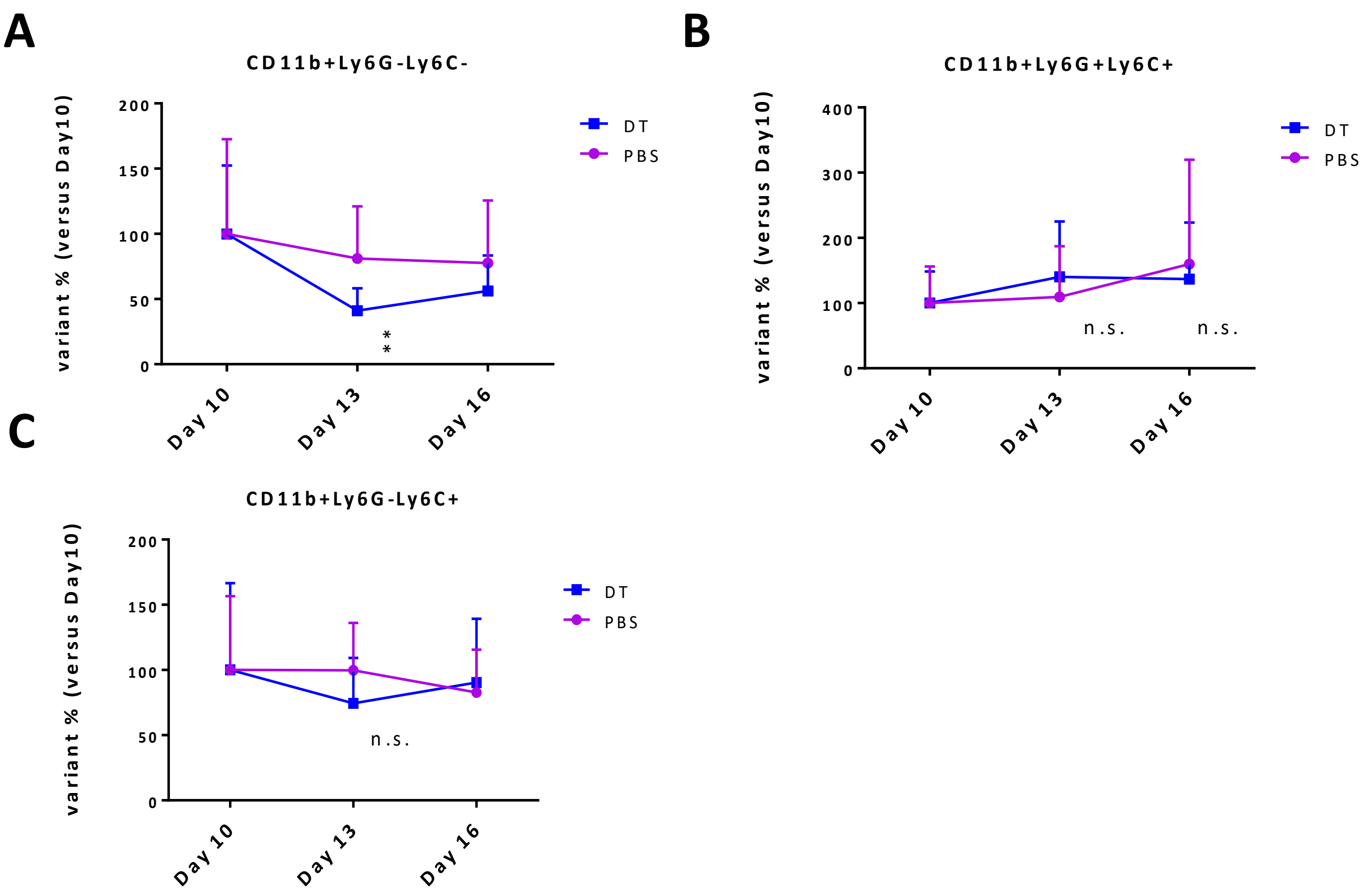
A
CD11b+Ly6G-Ly6C-
variant % (versus Day10)
200
150
100
50
0
Day 10
Day 13
Day 16
DT
PBS
*
*
B
CD11b+Ly6G+Ly6C+
variant % (versus Day10)
400
300
200
100
0
n.s.
n.s.
Day 10
Day 13
Day 16
DT
PBS
C
CD11b+Ly6G-Ly6C+
variant % (versus Day10)
200
150
100
50
0
n.s.
Day 10
Day 13
Day 16
DT
PBS

Figure S3

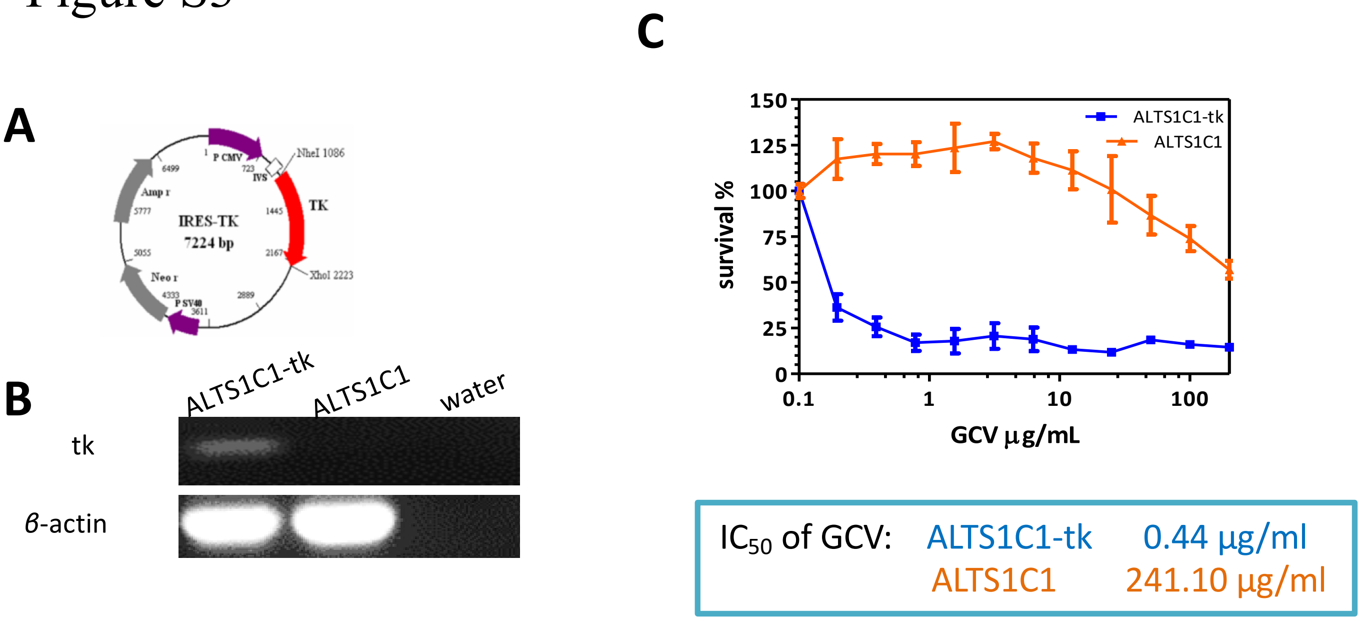

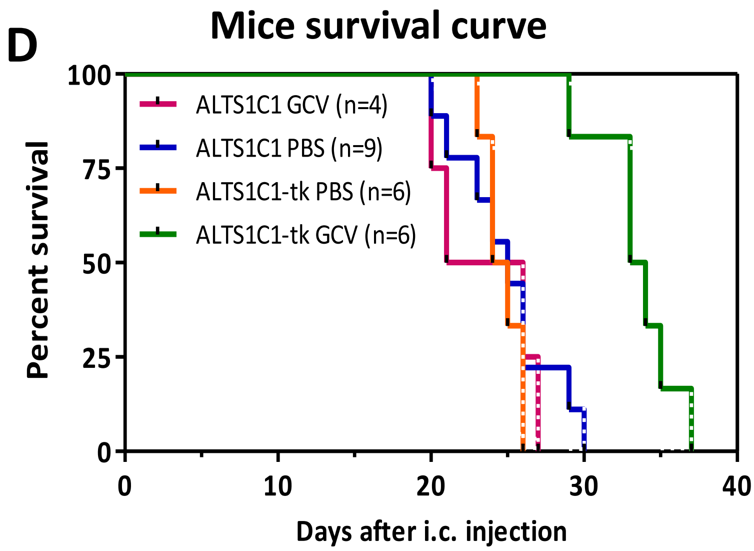

Group	Mean surviving day ± SD
ALTS1C1 GCV	23.5 ± 3.5
ALTS1C1 PBS	24.9 ± 3.3
ALTS1C1-tk PBS	24.7 ± 1.21
ALTS1C1-tk GCV	33.5 ± 2.67

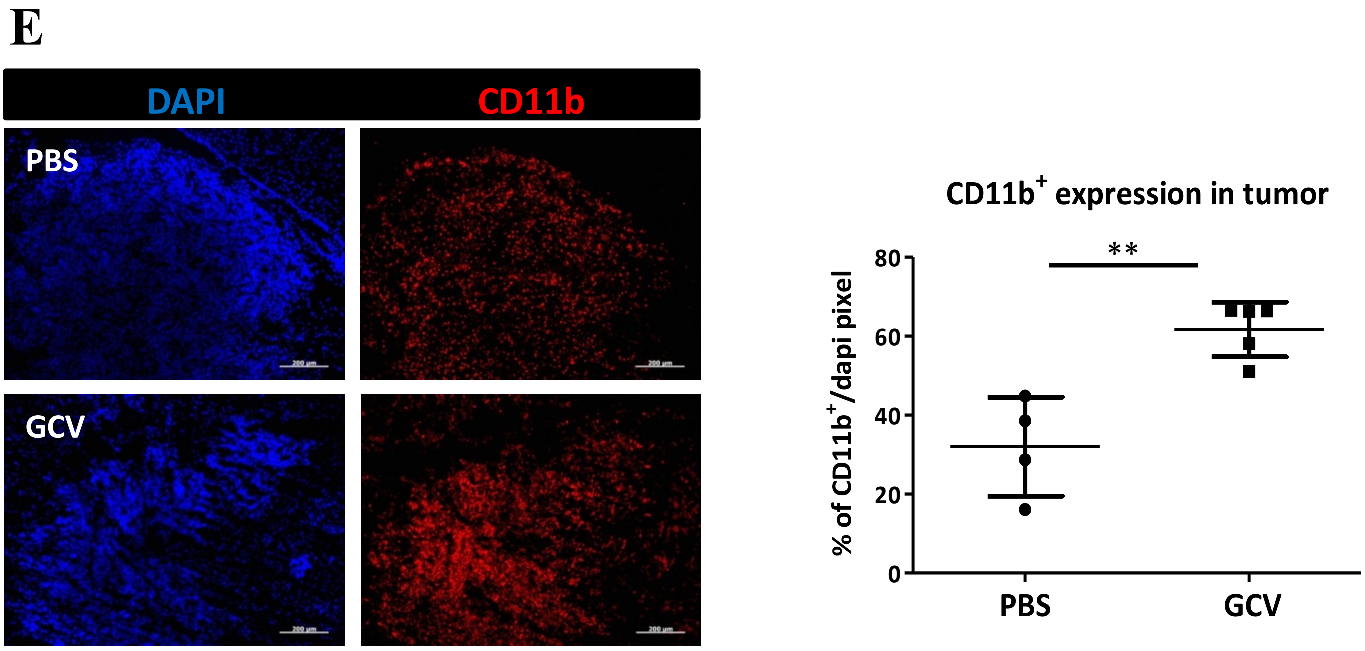

Supplement: Supplementary file 1 [file cells-09-00051-s001.pdf]
